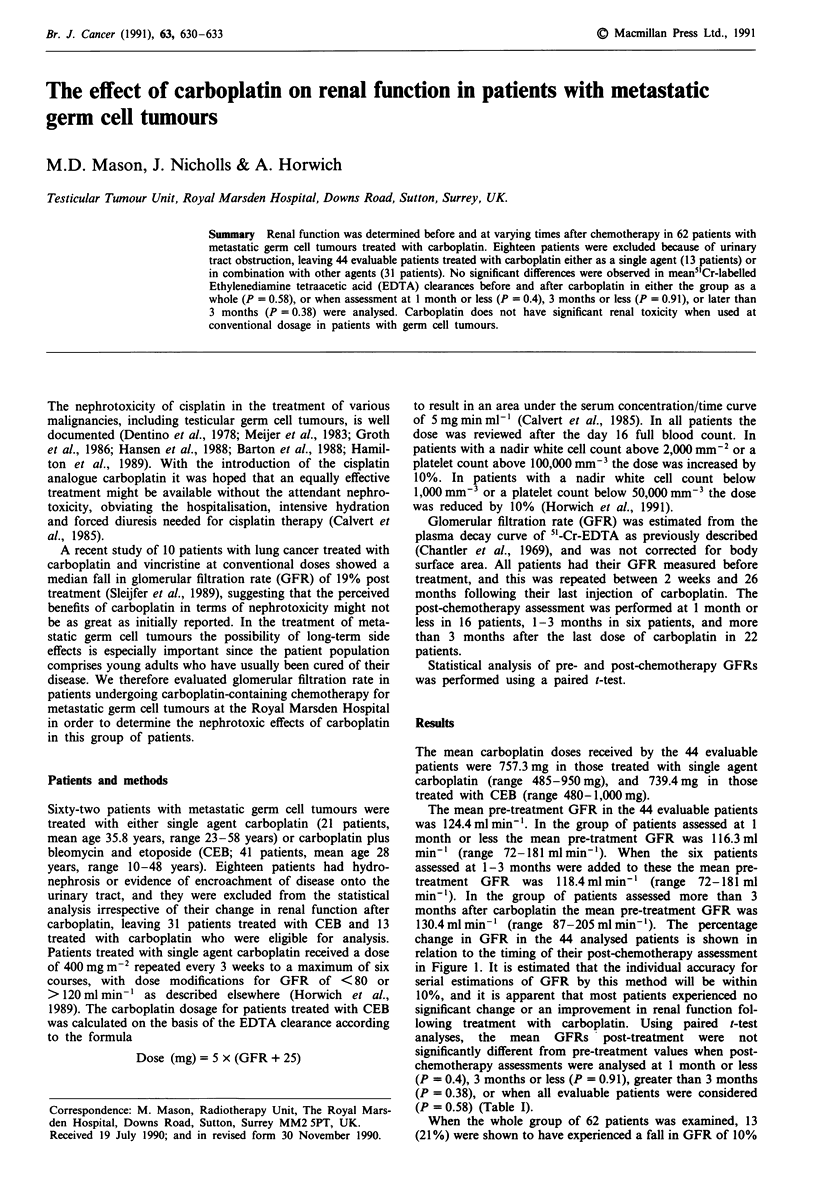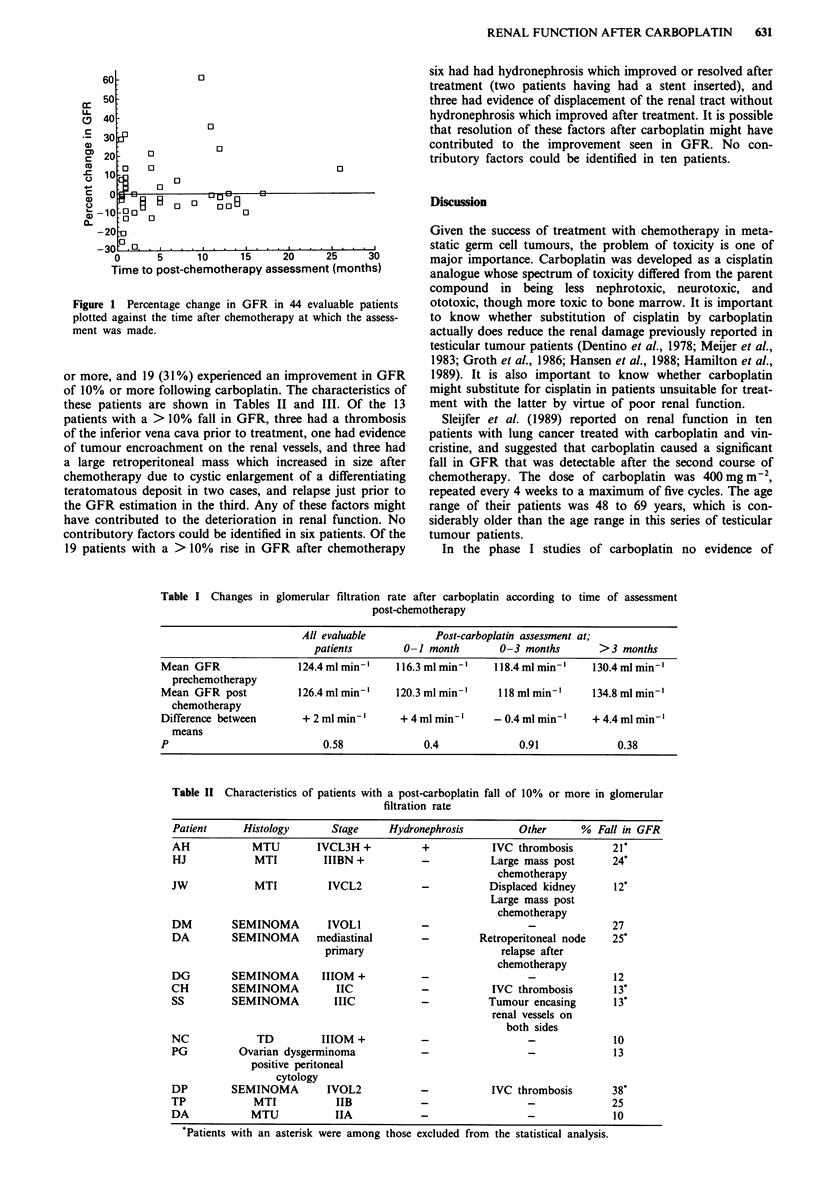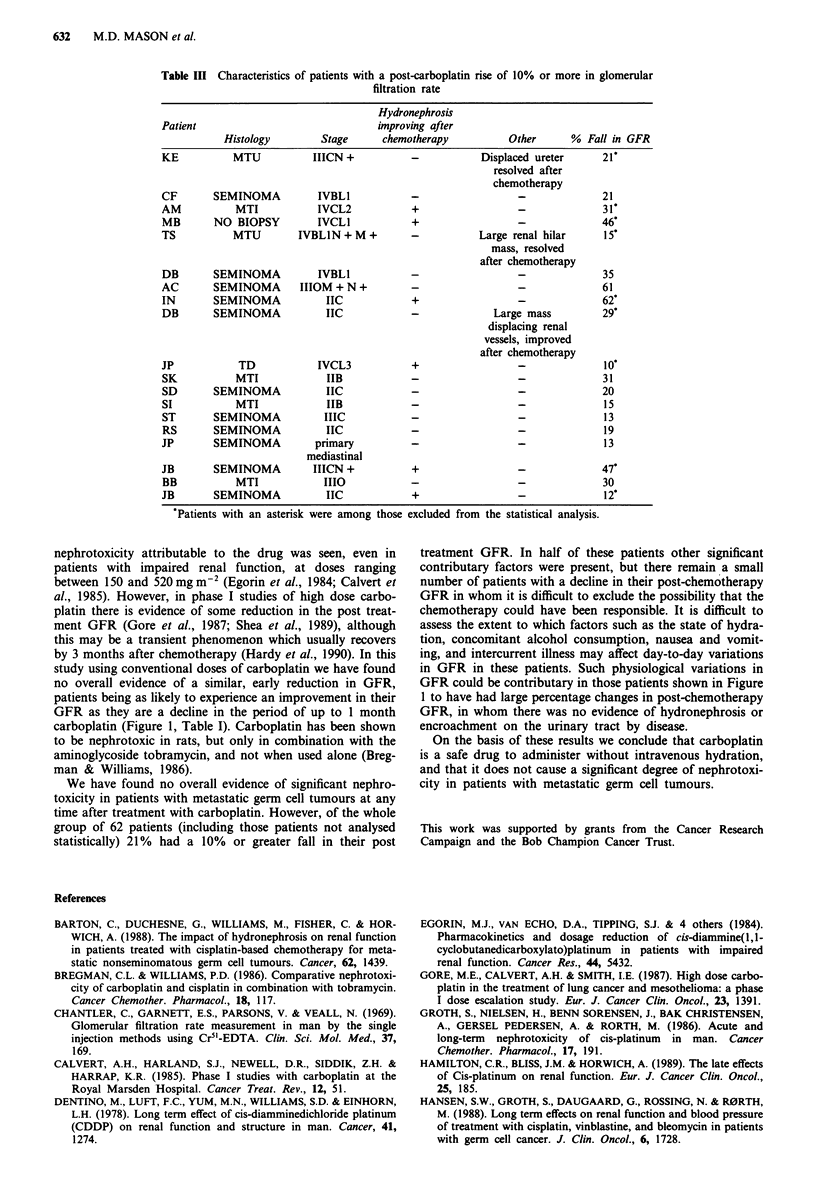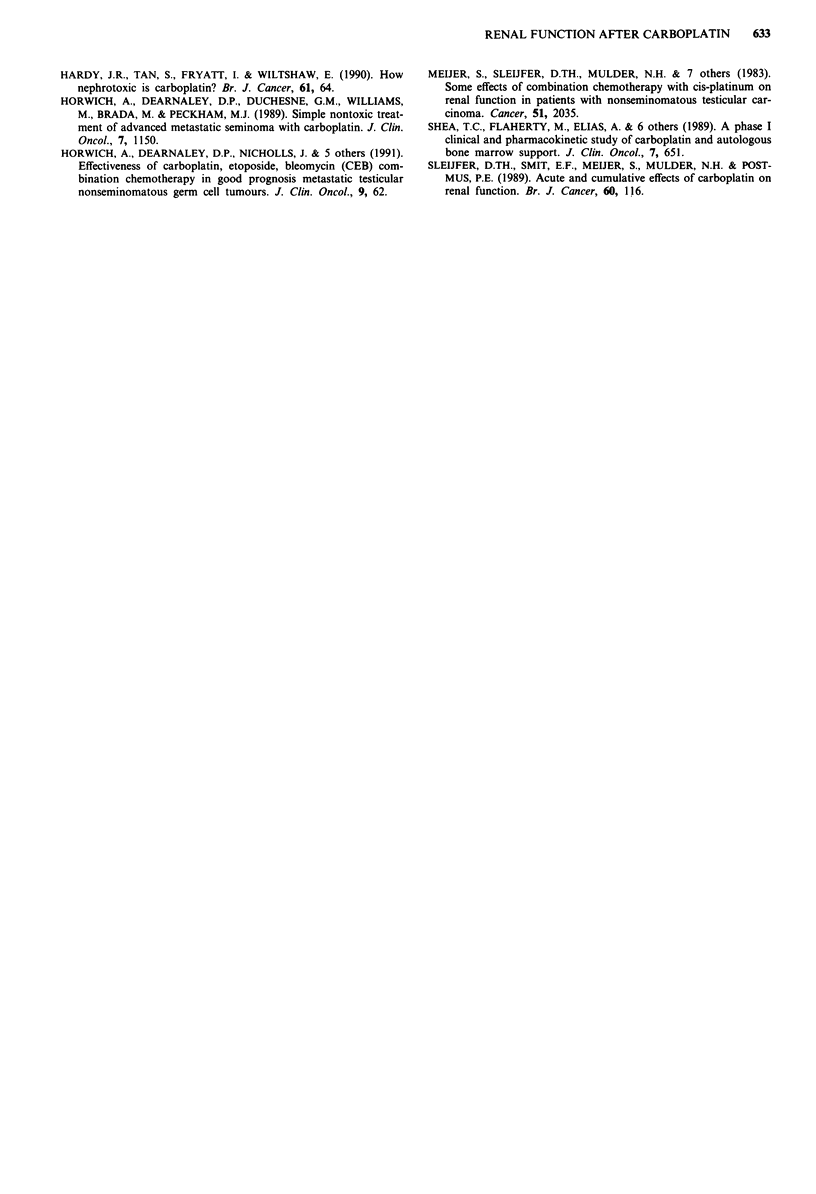# The effect of carboplatin on renal function in patients with metastatic germ cell tumours.

**DOI:** 10.1038/bjc.1991.144

**Published:** 1991-04

**Authors:** M. D. Mason, J. Nicholls, A. Horwich

**Affiliations:** Testicular Tumour Unit, Royal Marsden Hospital, Sutton, Surrey, UK.

## Abstract

Renal function was determined before and at varying times after chemotherapy in 62 patients with metastatic germ cell tumours treated with carboplatin. Eighteen patients were excluded because of urinary tract obstruction, leaving 44 evaluable patients treated with carboplatin either as a single agent (13 patients) or in combination with other agents (31 patients). No significant differences were observed in mean 51Cr-labelled Ethylenediamine tetraacetic acid (EDTA) clearances before and after carboplatin in either the group as a whole (P = 0.58), or when assessment at 1 month or less (P = 0.4), 3 months or less (P = 0.91), or later than 3 months (P = 0.38) were analysed. Carboplatin does not have significant renal toxicity when used at conventional dosage in patients with germ cell tumours.


 © Macmillan Press Ltd., 1991

### The effect of carboplatin on renal function in patients with metastatic germ cell tumours

M.D. Mason, J. Nicholls & A. Horwich

*Testicular Tumour Unit, Royal Marsden Hospital, Downs Road, Sutton, Surrey, UK.*

**Summary** Renal function was determined before and at varying times after chemotherapy in 62 patients with metastatic germ cell tumours treated with carboplatin. Eighteen patients were excluded because of urinary tract obstruction, leaving 44 evaluable patients treated with carboplatin either as a single agent (13 patients) or in combination with other agents (31 patients). No significant differences were observed in mean$^{51}$Cr-labelled Ethylenediamine tetraacetic acid (EDTA) clearances before and after carboplatin in either the group as a whole ($P = 0.58$), or when assessment at 1 month or less ($P = 0.4$), 3 months or less ($P = 0.91$), or later than 3 months ($P = 0.38$) were analysed. Carboplatin does not have significant renal toxicity when used at conventional dosage in patients with germ cell tumours.

The nephrotoxicity of cisplatin in the treatment of various malignancies, including testicular germ cell tumours, is well documented (Dentino *et al.*, 1978; Meijer *et al.*, 1983; Groth *et al.*, 1986; Hansen *et al.*, 1988; Barton *et al.*, 1988; Hamilton *et al.*, 1989). With the introduction of the cisplatin analogue carboplatin it was hoped that an equally effective treatment might be available without the attendant nephrotoxicity, obviating the hospitalisation, intensive hydration and forced diuresis needed for cisplatin therapy (Calvert *et al.*, 1985).

A recent study of 10 patients with lung cancer treated with carboplatin and vincristine at conventional doses showed a median fall in glomerular filtration rate (GFR) of 19% post treatment (Sleijfer *et al.*, 1989), suggesting that the perceived benefits of carboplatin in terms of nephrotoxicity might not be as great as initially reported. In the treatment of metastatic germ cell tumours the possibility of long-term side effects is especially important since the patient population comprises young adults who have usually been cured of their disease. We therefore evaluated glomerular filtration rate in patients undergoing carboplatin-containing chemotherapy for metastatic germ cell tumours at the Royal Marsden Hospital in order to determine the nephrotoxic effects of carboplatin in this group of patients.

### Patients and methods

Sixty-two patients with metastatic germ cell tumours were treated with either single agent carboplatin (21 patients, mean age 35.8 years, range 23-58 years) or carboplatin plus bleomycin and etoposide (CEB; 41 patients, mean age 28 years, range 10-48 years). Eighteen patients had hydronephrosis or evidence of encroachment of disease onto the urinary tract, and they were excluded from the statistical analysis irrespective of their change in renal function after carboplatin, leaving 31 patients treated with CEB and 13 treated with carboplatin who were eligible for analysis. Patients treated with single agent carboplatin received a dose of 400 mg $m^{-2}$ repeated every 3 weeks to a maximum of six courses, with dose modifications for GFR of $<80$ or $>120$ ml $min^{-1}$ as described elsewhere (Horwich *et al.*, 1989). The carboplatin dosage for patients treated with CEB was calculated on the basis of the EDTA clearance according to the formula

$$\text{Dose (mg)} = 5 \times (\text{GFR} + 25)$$

to result in an area under the serum concentration/time curve of 5 mg min $ml^{-1}$ (Calvert *et al.*, 1985). In all patients the dose was reviewed after the day 16 full blood count. In patients with a nadir white cell count above 2,000 $mm^{-2}$ or a platelet count above 100,000 $mm^{-3}$ the dose was increased by 10%. In patients with a nadir white cell count below 1,000 $mm^{-3}$ or a platelet count below 50,000 $mm^{-3}$ the dose was reduced by 10% (Horwich *et al.*, 1991).

Glomerular filtration rate (GFR) was estimated from the plasma decay curve of $^{51}$-Cr-EDTA as previously described (Chantler *et al.*, 1969), and was not corrected for body surface area. All patients had their GFR measured before treatment, and this was repeated between 2 weeks and 26 months following their last injection of carboplatin. The post-chemotherapy assessment was performed at 1 month or less in 16 patients, 1-3 months in six patients, and more than 3 months after the last dose of carboplatin in 22 patients.

Statistical analysis of pre- and post-chemotherapy GFRs was performed using a paired *t*-test.

### Results

The mean carboplatin doses received by the 44 evaluable patients were 757.3 mg in those treated with single agent carboplatin (range 485-950 mg), and 739.4 mg in those treated with CEB (range 480-1,000 mg).

The mean pre-treatment GFR in the 44 evaluable patients was 124.4 ml $min^{-1}$. In the group of patients assessed at 1 month or less the mean pre-tratment GFR was 116.3 ml $min^{-1}$ (range 72-181 ml $min^{-1}$). When the six patients assessed at 1-3 months were added to these the mean pre-treatment GFR was 118.4 ml $min^{-1}$ (range 72-181 ml $min^{-1}$). In the group of patients assessed more than 3 months after carboplatin the mean pre-treatment GFR was 130.4 ml $min^{-1}$ (range 87-205 ml $min^{-1}$). The percentage change in GFR in the 44 analysed patients is shown in relation to the timing of their post-chemotherapy assessment in Figure 1. It is estimated that the individual accuracy for serial estimations of GFR by this method will be within 10%, and it is apparent that most patients experienced no significant change or an improvement in renal function following treatment with carboplatin. Using paired *t*-test analyses, the mean GFRs post-treatment were not significantly different from pre-treatment values when post-chemotherapy assessments were analysed at 1 month or less ($P = 0.4$), 3 months or less ($P = 0.91$), greater than 3 months ($P = 0.38$), or when all evaluable patients were considered ($P = 0.58$) (Table I).

When the whole group of 62 patients was examined, 13 (21%) were shown to have experienced a fall in GFR of 10%

Correspondence: M. Mason, Radiotherapy Unit, The Royal Marsden Hospital, Downs Road, Sutton, Surrey MM2 5PT, UK.
Received 19 July 1990; and in revised form 30 November 1990.

**Figure 1** Percentage change in GFR in 44 evaluable patients plotted against the time after chemotherapy at which the assessment was made.

or more, and 19 (31%) experienced an improvement in GFR of 10% or more following carboplatin. The characteristics of these patients are shown in Tables II and III. Of the 13 patients with a >10% fall in GFR, three had a thrombosis of the inferior vena cava prior to treatment, one had evidence of tumour encroachment on the renal vessels, and three had a large retroperitoneal mass which increased in size after chemotherapy due to cystic enlargement of a differentiating teratomatous deposit in two cases, and relapse just prior to the GFR estimation in the third. Any of these factors might have contributed to the deterioration in renal function. No contributory factors could be identified in six patients. Of the 19 patients with a >10% rise in GFR after chemotherapy six had had hydronephrosis which improved or resolved after treatment (two patients having had a stent inserted), and three had evidence of displacement of the renal tract without hydronephrosis which improved after treatment. It is possible that resolution of these factors after carboplatin might have contributed to the improvement seen in GFR. No contributory factors could be identified in ten patients.

### Discussion

Given the success of treatment with chemotherapy in metastatic germ cell tumours, the problem of toxicity is one of major importance. Carboplatin was developed as a cisplatin analogue whose spectrum of toxicity differed from the parent compound in being less nephrotoxic, neurotoxic, and ototoxic, though more toxic to bone marrow. It is important to know whether substitution of cisplatin by carboplatin actually does reduce the renal damage previously reported in testicular tumour patients (Dentino *et al.*, 1978; Meijer *et al.*, 1983; Groth *et al.*, 1986; Hansen *et al.*, 1988; Hamilton *et al.*, 1989). It is also important to know whether carboplatin might substitute for cisplatin in patients unsuitable for treatment with the latter by virtue of poor renal function.

Sleijfer *et al.* (1989) reported on renal function in ten patients with lung cancer treated with carboplatin and vincristine, and suggested that carboplatin caused a significant fall in GFR that was detectable after the second course of chemotherapy. The dose of carboplatin was 400 mg $m^{-2}$, repeated every 4 weeks to a maximum of five cycles. The age range of their patients was 48 to 69 years, which is considerably older than the age range in this series of testicular tumour patients.

In the phase I studies of carboplatin no evidence of

**Table I** Changes in glomerular filtration rate after carboplatin according to time of assessment post-chemotherapy

| | *All evaluable patients* | *Post-carboplatin assessment at; 0–1 month* | *0–3 months* | *>3 months* |
|---|---|---|---|---|
| Mean GFR prechemotherapy | 124.4 ml $min^{-1}$ | 116.3 ml $min^{-1}$ | 118.4 ml $min^{-1}$ | 130.4 ml $min^{-1}$ |
| Mean GFR post chemotherapy | 126.4 ml $min^{-1}$ | 120.3 ml $min^{-1}$ | 118 ml $min^{-1}$ | 134.8 ml $min^{-1}$ |
| Difference between means | + 2 ml $min^{-1}$ | + 4 ml $min^{-1}$ | − 0.4 ml $min^{-1}$ | + 4.4 ml $min^{-1}$ |
| *P* | 0.58 | 0.4 | 0.91 | 0.38 |

**Table II** Characteristics of patients with a post-carboplatin fall of 10% or more in glomerular filtration rate

| *Patient* | *Histology* | *Stage* | *Hydronephrosis* | *Other* | *% Fall in GFR* |
|---|---|---|---|---|---|
| AH | MTU | IVCL3H + | + | IVC thrombosis | 21* |
| HJ | MTI | IIIBN + | − | Large mass post chemotherapy | 24* |
| JW | MTI | IVCL2 | − | Displaced kidney Large mass post chemotherapy | 12* |
| DM | SEMINOMA | IVOL1 | − | − | 27 |
| DA | SEMINOMA | mediastinal primary | − | Retroperitoneal node relapse after chemotherapy | 25* |
| DG | SEMINOMA | IIIOM + | − | − | 12 |
| CH | SEMINOMA | IIC | − | IVC thrombosis | 13* |
| SS | SEMINOMA | IIIC | − | Tumour encasing renal vessels on both sides | 13* |
| NC | TD | IIIOM + | − | − | 10 |
| PG | Ovarian dysgerminoma positive peritoneal cytology | | − | − | 13 |
| DP | SEMINOMA | IVOL2 | − | IVC thrombosis | 38* |
| TP | MTI | IIB | − | − | 25 |
| DA | MTU | IIA | − | − | 10 |

*Patients with an asterisk were among those excluded from the statistical analysis.

**Table III** Characteristics of patients with a post-carboplatin rise of 10% or more in glomerular filtration rate

| *Patient* | *Histology* | *Stage* | *Hydronephrosis improving after chemotherapy* | *Other* | *% Fall in GFR* |
|---|---|---|---|---|---|
| KE | MTU | IIICN + | – | Displaced ureter resolved after chemotherapy | 21* |
| CF | SEMINOMA | IVBL1 | – | – | 21 |
| AM | MTI | IVCL2 | + | – | 31* |
| MB | NO BIOPSY | IVCL1 | + | – | 46* |
| TS | MTU | IVBL1N + M + | – | Large renal hilar mass, resolved after chemotherapy | 15* |
| DB | SEMINOMA | IVBL1 | – | – | 35 |
| AC | SEMINOMA | IIIOM + N + | – | – | 61 |
| IN | SEMINOMA | IIC | + | – | 62* |
| DB | SEMINOMA | IIC | – | Large mass displacing renal vessels, improved after chemotherapy | 29* |
| JP | TD | IVCL3 | + | – | 10* |
| SK | MTI | IIB | – | – | 31 |
| SD | SEMINOMA | IIC | – | – | 20 |
| SI | MTI | IIB | – | – | 15 |
| ST | SEMINOMA | IIIC | – | – | 13 |
| RS | SEMINOMA | IIC | – | – | 19 |
| JP | SEMINOMA | primary mediastinal | – | – | 13 |
| JB | SEMINOMA | IIICN + | + | – | 47* |
| BB | MTI | IIIO | – | – | 30 |
| JB | SEMINOMA | IIC | + | – | 12* |

*Patients with an asterisk were among those excluded from the statistical analysis.

nephrotoxicity attributable to the drug was seen, even in patients with impaired renal function, at doses ranging between 150 and 520 mg $m^{-2}$ (Egorin *et al.*, 1984; Calvert *et al.*, 1985). However, in phase I studies of high dose carboplatin there is evidence of some reduction in the post treatment GFR (Gore *et al.*, 1987; Shea *et al.*, 1989), although this may be a transient phenomenon which usually recovers by 3 months after chemotherapy (Hardy *et al.*, 1990). In this study using conventional doses of carboplatin we have found no overall evidence of a similar, early reduction in GFR, patients being as likely to experience an improvement in their GFR as they are a decline in the period of up to 1 month carboplatin (Figure 1, Table I). Carboplatin has been shown to be nephrotoxic in rats, but only in combination with the aminoglycoside tobramycin, and not when used alone (Bregman & Williams, 1986).

We have found no overall evidence of significant nephrotoxicity in patients with metastatic germ cell tumours at any time after treatment with carboplatin. However, of the whole group of 62 patients (including those patients not analysed statistically) 21% had a 10% or greater fall in their post treatment GFR. In half of these patients other significant contributary factors were present, but there remain a small number of patients with a decline in their post-chemotherapy GFR in whom it is difficult to exclude the possibility that the chemotherapy could have been responsible. It is difficult to assess the extent to which factors such as the state of hydration, concomitant alcohol consumption, nausea and vomiting, and intercurrent illness may affect day-to-day variations in GFR in these patients. Such physiological variations in GFR could be contributary in those patients shown in Figure 1 to have had large percentage changes in post-chemotherapy GFR, in whom there was no evidence of hydronephrosis or encroachment on the urinary tract by disease.

On the basis of these results we conclude that carboplatin is a safe drug to administer without intravenous hydration, and that it does not cause a significant degree of nephrotoxicity in patients with metastatic germ cell tumours.

This work was supported by grants from the Cancer Research Campaign and the Bob Champion Cancer Trust.

### References

BARTON, C., DUCHESNE, G., WILLIAMS, M., FISHER, C. & HORWICH, A. (1988). The impact of hydronephrosis on renal function in patients treated with cisplatin-based chemotherapy for metastatic nonseminomatous germ cell tumours. *Cancer*, **62**, 1439.

BREGMAN, C.L. & WILLIAMS, P.D. (1986). Comparative nephrotoxicity of carboplatin and cisplatin in combination with tobramycin. *Cancer Chemother. Pharmacol.*, **18**, 117.

CHANTLER, C., GARNETT, E.S., PARSONS, V. & VEALL, N. (1969). Glomerular filtration rate measurement in man by the single injection methods using $Cr^{51}$-EDTA. *Clin. Sci. Mol. Med.*, **37**, 169.

CALVERT, A.H., HARLAND, S.J., NEWELL, D.R., SIDDIK, Z.H. & HARRAP, K.R. (1985). Phase I studies with carboplatin at the Royal Marsden Hospital. *Cancer Treat. Rev.*, **12**, 51.

DENTINO, M., LUFT, F.C., YUM, M.N., WILLIAMS, S.D. & EINHORN, L.H. (1978). Long term effect of cis-diamminedichloride platinum (CDDP) on renal function and structure in man. *Cancer*, **41**, 1274.

EGORIN, M.J., VAN ECHO, D.A., TIPPING, S.J. & 4 others (1984). Pharmacokinetics and dosage reduction of *cis*-diammine(1,1-cyclobutanedicarboxylato)platinum in patients with impaired renal function. *Cancer Res.*, **44**, 5432.

GORE, M.E., CALVERT, A.H. & SMITH, I.E. (1987). High dose carboplatin in the treatment of lung cancer and mesothelioma: a phase I dose escalation study. *Eur. J. Cancer Clin. Oncol.*, **23**, 1391.

GROTH, S., NIELSEN, H., BENN SORENSEN, J., BAK CHRISTENSEN, A., GERSEL PEDERSEN, A. & RORTH, M. (1986). Acute and long-term nephrotoxicity of cis-platinum in man. *Cancer Chemother. Pharmacol.*, **17**, 191.

HAMILTON, C.R., BLISS, J.M. & HORWICH, A. (1989). The late effects of Cis-platinum on renal function. *Eur. J. Cancer Clin. Oncol.*, **25**, 185.

HANSEN, S.W., GROTH, S., DAUGAARD, G., ROSSING, N. & RØRTH, M. (1988). Long term effects on renal function and blood pressure of treatment with cisplatin, vinblastine, and bleomycin in patients with germ cell cancer. *J. Clin. Oncol.*, **6**, 1728.

HARDY, J.R., TAN, S., FRYATT, I. & WILTSHAW, E. (1990). How nephrotoxic is carboplatin? *Br. J. Cancer*, **61**, 64.

HORWICH, A., DEARNALEY, D.P., DUCHESNE, G.M., WILLIAMS, M., BRADA, M. & PECKHAM, M.J. (1989). Simple nontoxic treatment of advanced metastatic seminoma with carboplatin. *J. Clin. Oncol.*, **7**, 1150.

HORWICH, A., DEARNALEY, D.P., NICHOLLS, J. & 5 others (1991). Effectiveness of carboplatin, etoposide, bleomycin (CEB) combination chemotherapy in good prognosis metastatic testicular nonseminomatous germ cell tumours. *J. Clin. Oncol.*, **9**, 62.

MEIJER, S., SLEIJFER, D.TH., MULDER, N.H. & 7 others (1983). Some effects of combination chemotherapy with cis-platinum on renal function in patients with nonseminomatous testicular carcinoma. *Cancer*, **51**, 2035.

SHEA, T.C., FLAHERTY, M., ELIAS, A. & 6 others (1989). A phase I clinical and pharmacokinetic study of carboplatin and autologous bone marrow support. *J. Clin. Oncol.*, **7**, 651.

SLEIJFER, D.TH., SMIT, E.F., MEIJER, S., MULDER, N.H. & POSTMUS, P.E. (1989). Acute and cumulative effects of carboplatin on renal function. *Br. J. Cancer*, **60**, 116.